# A Noise Filtering Algorithm for Event-Based Asynchronous Change Detection Image Sensors on TrueNorth and Its Implementation on TrueNorth

**DOI:** 10.3389/fnins.2018.00118

**Published:** 2018-03-05

**Authors:** Vandana Padala, Arindam Basu, Garrick Orchard

**Affiliations:** ^1^School of Electrical and Electronic Engineering, Nanyang Technological University, Singapore, Singapore; ^2^Singapore Institute for Neurotechnology (SINAPSE), National University of Singapore, Singapore, Singapore; ^3^Temasek Labs, National University of Singapore, Singapore, Singapore

**Keywords:** TrueNorth, neuromorphic vision, noise filtering, event based camera, silicon retina, neural network

## Abstract

Asynchronous event-based sensors, or “silicon retinae,” are a new class of vision sensors inspired by biological vision systems. The output of these sensors often contains a significant number of noise events along with the signal. Filtering these noise events is a common preprocessing step before using the data for tasks such as tracking and classification. This paper presents a novel spiking neural network-based approach to filtering noise events from data captured by an Asynchronous Time-based Image Sensor on a neuromorphic processor, the IBM TrueNorth Neurosynaptic System. The significant contribution of this work is that it demonstrates our proposed filtering algorithm outperforms the traditional nearest neighbor noise filter in achieving higher signal to noise ratio (~10 dB higher) and retaining the events related to signal (~3X more). In addition, for our envisioned application of object tracking and classification under some parameter settings, it can also generate some of the missing events in the spatial neighborhood of the signal for all classes of moving objects in the data which are unattainable using the nearest neighbor filter.

## 1. Introduction

Inspired by the efficient operation of biological vision, research on neuromorphic event-based image sensors, or “silicon retinae,” took off a few decades back (Mahowald and Mead, 1991). Recently, the technology has matured to a point where the sensors are commercially available. Dynamic Vision Sensor (DVS) (Lichtsteiner et al., 2008), Asynchronous Time-based Image Sensor (ATIS) (Posch et al., 2011), the sensitive DVS (Leñero-Bardallo et al., 2011), and the Dynamic and Active pixel Vision Sensor (DAVIS) (Berner et al., 2013) are some of the popular Address Event Representation (AER) change detection sensors that can be employed for various applications. Unlike conventional image sensors that operate by sampling the scene at a fixed temporal rate (typically between 30 and 60 Hz), these sensors employ level crossing sampling pixels which asynchronously and independently signal an event if sufficient temporal contrast is detected (Posch et al., 2014). This results in a higher dynamic range, lower data rate and lower power consumption compared to frame based imagers. Several possible applications of these sensors have been investigated including traffic monitoring (Litzenberger et al., 2006), stereovision (Rogister et al., 2012), high speed sensory-motor loops (Delbruck and Lang, 2013), motion estimation (Orchard and Etienne-Cummings, 2015) and pose estimation (Valeiras et al., 2016).

However, most works on processing the events generated by silicon retina are performed on FPGA or microcontrollers resulting in lower power efficiency of the whole system than that afforded by the sensors. Hence, interfacing these sensors with low-power neuromorphic processors would benefit the development of low power systems that could potentially be deployed in wearables or as sensors in the Internet of Things (IoT). Several low power neuromorphic processors have been proposed in recent years (Painkras et al., 2013; Benjamin et al., 2014; Chen et al., 2016) and some of them have been interfaced with spiking image sensors (Orchard et al., 2015). However, the power dissipation of these systems are still an order of magnitude more than that required for a wearable device. On the other hand, the TrueNorth processor developed by IBM (Merolla et al., 2014) has both the low power footprint and the large neuron count available on a single chip required to interface with image sensors. It processes spiking data in real-time with a fixed 1 kHz clock using distributed memory kept locally in each core instead of one central location. TrueNorth consumes very low power of approximately 50–70 mW for typical networks and results in a good combination of power efficiency and configurability. As a consequence, there have been recent reports of interfacing the TrueNorth with “silicon retinae” to create real-time low-power vision applications (Amir et al., 2017).

In keeping with the above trend, we propose in this work a set of noise filtering primitives that may be used as a pre-processing block for event-based image processing applications on TrueNorth. The concepts presented in this paper can also be used to implement noise filtering for silicon retinae on other embedded hardware platforms. In this work, we focus on the case of a static camera observing a scene such as in a surveillance scenario. Noise filtering algorithms such as nearest neighbor for event-based imagers presented in the literature exploit the temporal correlation associated with activity across the neighborhood pixels (Dominguez-Morales, 2011; Ieng et al., 2014; Linares-Barranco et al., 2015; Liu et al., 2015; Czech and Orchard, 2016). Though these filters capture the activity of the fast moving objects, they typically filter out the activity by the small and slow-moving objects due to weak temporal support. The spiking neural network based noise filter proposed in this paper is shown to work better than other popular filters in this respect and preserve a large fraction of activity associated with the signal (and sometimes generate more events than input) while filtering out most of the noise events which is beneficial to object tracking and classification in our envisioned applications.

This paper is structured as follows. In section 2, we describe the ATIS setup used in our experiments as well as the nature of data recorded. Section 3 provides an overview of TrueNorth, while in section 4, we introduce the noise filtering approaches. We present the implementation of the noise filter on TrueNorth in section 5 and the results of the experiments are presented in section 6. Finally, we conclude the paper with some discussions about future work in section 7.

## 2. Asynchronous event based image sensor (ATIS)

Unlike the traditional frame-based computer vision sensors which capture the absolute light intensity information (grayscale or color pixel values) at constant time intervals (frame-period), AER change detection image sensors operate by detecting when and where the intensity changes are occurring in the scene. In this work, we use ATIS which has a resolution of 304 × 240 = 72,960 pixels.The output of the ATIS data is represented in the form of a stream of events in the AER format. In the AER format, the *k*^*th*^ event, *e*_*k*_, consists of the physical address (*x*_*k*_,*y*_*k*_) of the pixel which generated the event, time stamp, *t*_*k*_, of the event (in microseconds) and the polarity, *p*_*k*_, representing whether the intensity change is in positive or negative direction, i.e., *p*_*k*_ ∈ {1, −1}. In the mathematical form of this event, *e*_*k*_ is represented as follows:

(1) $$\begin{array}{r} e_{k}=\left(x_{k},y_{k},t_{k},p_{k}\right) \end{array}$$

The description of event generation mechanism, in-depth details and the working of the internal circuitry of various event-based sensors is presented in Posch et al. (2014).

### 2.1. ATIS data

**Figure 1:**
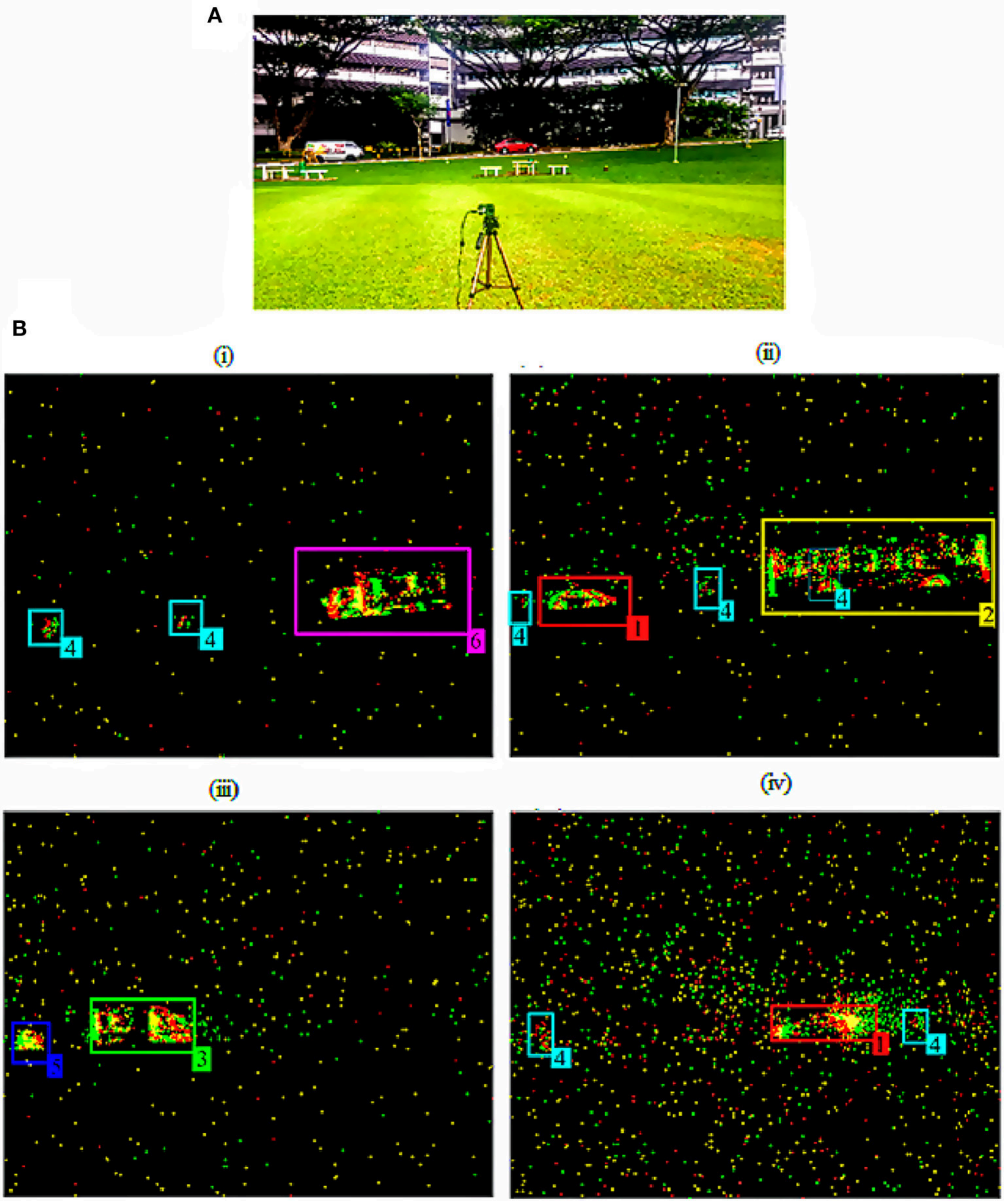
Data Recording from the ATIS setup. **(A)** ATIS setup to record the moving vehicles and **(B)** Four Screenshots at (i) 111.78 s from 6:00 p.m. recording, (ii) 39.67 s from 6:30 p.m. recording, (iii) 34.29 s from 6:50 p.m. recording and (iv) 34.48 s from 7:15 p.m. recording showing the moving objects captured by the ATIS in **(A)** in the scene with six different types of objects (1) Car (Red), (2) Bus (Yellow), (3) Van (Green), (4) Human (Cyan), (5) Bike (Blue), and (6) Truck (Pink) indicated with respective label number and color tracker mentioned in Table 2.

The ATIS setup at a traffic junction used to acquire AER data of the moving objects on the road from the side view is shown in Figure 1A. The moving entities in the data fall into six categories: (1) Car, (2) Bus, (3) Van, (4) Pedestrian (Human), (5) Bike, and (6) Truck. There are 9 sample recordings of varying duration and a varying number of these objects, capturing the movement of pedestrians and vehicles on the road in the field of view from this camera setup and at a fixed distance of approximately 50 m. Each recording has a different amount of daylight starting from the first recording at 6:00 p.m. to the last one at around 7:15 p.m. Comprehensive details of these recordings are provided in Tables 1, 2.

**Table 1:** Details of the traffic data recorded using ATIS.

**Recording start time**	**Recording length (s)**	**Tracks**	**Car**	**Bus**	**Van**	**Human**	**Bike**	**Truck**	**Average noise activity [(events per pixel)/sec]**
6:00 p.m.	113.4332	33	19	2	1	6	2	3	0.2760
6:10 p.m.	149.2754	46	29	4	0	11	2	0	0.2996
6:30 p.m.	123.6664	35	16	4	0	14	0	1	0.3755
6:35 p.m.	153.8343	38	27	2	1	6	2	0	0.3933
6:45 p.m.	138.3563	41	29	4	2	3	3	0	0.6463
6:50 p.m.	90.2431	38	26	0	3	3	6	0	0.7881
7:00 p.m.	117.7797	35	19	4	5	2	5	0	1.5035
7:05 p.m.	92.3087	23	14	1	0	7	1	0	1.6560
7:15 p.m.	143.4216	40	32	1	2	5	0	0	1.8294

**Table 2:** Details of each class of object present in the traffic data files.

**Label No**	**1**	**2**	**3**	**4**	**5**	**6**
Tracker color	Red	Yellow	Green	Cyan	Blue	Magenta
Class	Car	Bus	Van	Pedestrian	Bike	Truck
Total objects in each class	211	22	14	57	21	4
Range of tracker width (in pixels)	40–95	90–200	50-110	8-26	15–42	65–150
Range of tracker height (in pixels)	16–35	26–125	20-45	12-36	16–35	30–60
Average tracker width (in pixels)	62	142	68	16	29	112
Average tracker height (in pixels)	24	54	32	22	26	47
Avg. no of events/track	69,942	364,738	145,969	36,795	38,820	169,805
Total events in each class	14,757,904	8,024,246	2,043,578	2,097,337	815,226	679,222
Total track time (in sec)	83.3188	11.0117	6.8088	112.8199	7.6030	1.7351
Spike rate (spikes/sec)	177,130	728,700	300,140	18,590	107,220	391,450
Average Area of tracker (average no. pixels)	1,488	7,668	2,176	352	754	5,264
Spike rate per pixel (per second)	119	95	138	53	142	74

The screenshots of the ATIS video output at various time instances from the four different recordings are presented in Figure 1B. These screenshots provide a glimpse of how objects were captured by the ATIS with varying background activity over time. We can also notice from the ATIS setup in Figure 1A and the screenshots of the recordings in Figure 1B that the activity of the objects captured primarily lies in the middle of the scene along the horizontal direction where objects are moving from right to left and left to right. For visual clarity, each object of interest in those screenshots is shown with a particular label/class number and color of the tracker it belongs to as mentioned in Table 2.

Annotation files for all the recordings were generated manually by observing the video outputs. They capture information about the track of each object in these recordings belonging to a particular class. The tracker (or the bounding box) of varying size surrounding the object is devised to encapsulate the event stream generated for each moving object track. Using the information of each tracker in the scene from the acquired data and the annotation files, we summarized the details of bounding box size and average events captured in each class of objects in the recordings in Table 2.

### 2.2. Data characteristics

From Table 1, it can be seen that the recordings fall under three groups depending on the frequency of object occurrence in the scene. The first group consists of most frequent appearing objects, which in our case is cars. The second group is buses, pedestrians and bikes whose frequency of appearance is less compared to that of cars. The last group is Van and Truck that appear even fewer times. These recordings have a number of non-idealities typically present in outdoor scene recordings. The characteristics of the events generated by the fast moving objects like a car, bus and bike differ significantly from the slow moving pedestrians. Often, it is challenging to preserve the activity of the slow moving pedestrians compared to the background noise during the noise filtering. Also, compactness of the vehicle (bike), contrast of the moving vehicle across its length (bus), shape of the vehicle (Van vs. Car and Bus vs. Truck) are some of the factors which determine how the moving object is captured in these recordings. These recordings also have a tree and two electric poles which occlude some part of the vehicle while moving through that location.

### 2.3. Signal and noise definitions

In this study, since our interest lies in extracting the moving objects, **Signal** is defined as the events captured in each tracker/bounding box surrounding the moving object. **Noise** is considered as the events or activity present outside this tracker/bounding box. Any noise filter acts on both the signal events which captures the moving object and the noise events present outside the bounding box of the object tracker. The task of filtering away the unwanted noise outside the tracker is an important pre-processing step in order to extract the objects for the classification task. A good filter retains most of the signal and removes most of the noise events present outside the tracker effectively after the filtering.

The sensor not only captures the change in the light intensity at a location due to moving objects but also produces some noise activity throughout the scene at various pixels due to the movements of background objects like trees and also due to the sensor noise. The presence of this noise activity in the background is a challenge for extracting objects for tracking. Depending on the time of recording, as illumination reduces, the relative strength of background activity increases as shown in Figure 1B and last column of Table 1. In the rest of the paper, we describe a neural network and its hardware implementation to filter out noise in scenes with such a variety of objects.

### 2.4. Evaluation metric

In order to quantify the noise filtering performance using the algorithms detailed in section 4 and their implementation on TrueNorth described in section 5 (Table 3), we first define the following terms and the metrics below to compute them on the unfiltered and filtered data.

**Table 3:** Definition and notation of the variables used in the metrics calculation.

**Variable name**	**Notation**
Total no. of events inside all the trackers of sample data before filtering	$E_{Signal}^{o}$
Total no. of events outside all the trackers of sample data before filtering	$E_{Noise}^{o}$
Total no of events inside all the trackers of sample data after applying the filter	$E_{Signal}^{f}$
Total no of events outside all the trackers of sample data after applying the filter	$E_{Noise}^{f}$
Total events inside all trackers of a particular class for all data files before applying the filter	$E_{class}^{o}$
Total events inside all trackers of a particular class for all data files after applying the filter	$E_{class}^{f}$

- **Percentage of Signal Remaining**

The percentage of signal remaining after filtering, *PSR*^*f*^, is defined as the number of the signal events after filtering with respect to the original number of signal events before filtering, i.e.,

(2) $$\begin{array}{r} {PSR}^{f}=\frac{E_{signal}^{f}}{E_{signal}^{o}}\times 100 \end{array}$$

- **Percentage of Noise Remaining**

The percentage noise remaining after filtering, *PNR*^*f*^, is defined as the number of noise events post-filtering as a percentage of number of noise events pre-filtering, i.e.,

(3) $$\begin{array}{r} {PNR}^{f}=\frac{E_{Noise}^{f}}{E_{Noise}^{o}}\times 100 \end{array}$$

- **Signal to Noise Ratio**

Signal to Noise Ratio before filtering:

(4) $$\begin{array}{r} {SNR}^{o}=10\times \log_{10}\left(\frac{E_{Signal}^{o}}{E_{Noise}^{o}}\right)\text{in Decibels} \end{array}$$

Signal to Noise Ratio after filtering:

(5) $$\begin{array}{r} {SNR}^{f}=10\times \log_{10}\left(\frac{E_{Signal}^{f}}{E_{Noise}^{f}}\right)\text{in Decibels} \end{array}$$

- **Percentage of Average number of Events remaining Per Class**

The percentage of average number of events remaining after filtering per class, ${EPC}_{class}^{f}$, is defined as the percentage of the total no of events after filtering of a particular class of object with respect to the original number of events of that class before filtering, i.e.:

(6) $$\begin{array}{r} {EPC}_{class}^{f}=\frac{E_{class}^{f}}{E_{class}^{o}}\times 100 \end{array}$$

## 3. TrueNorth: an overview

**Figure 2:**
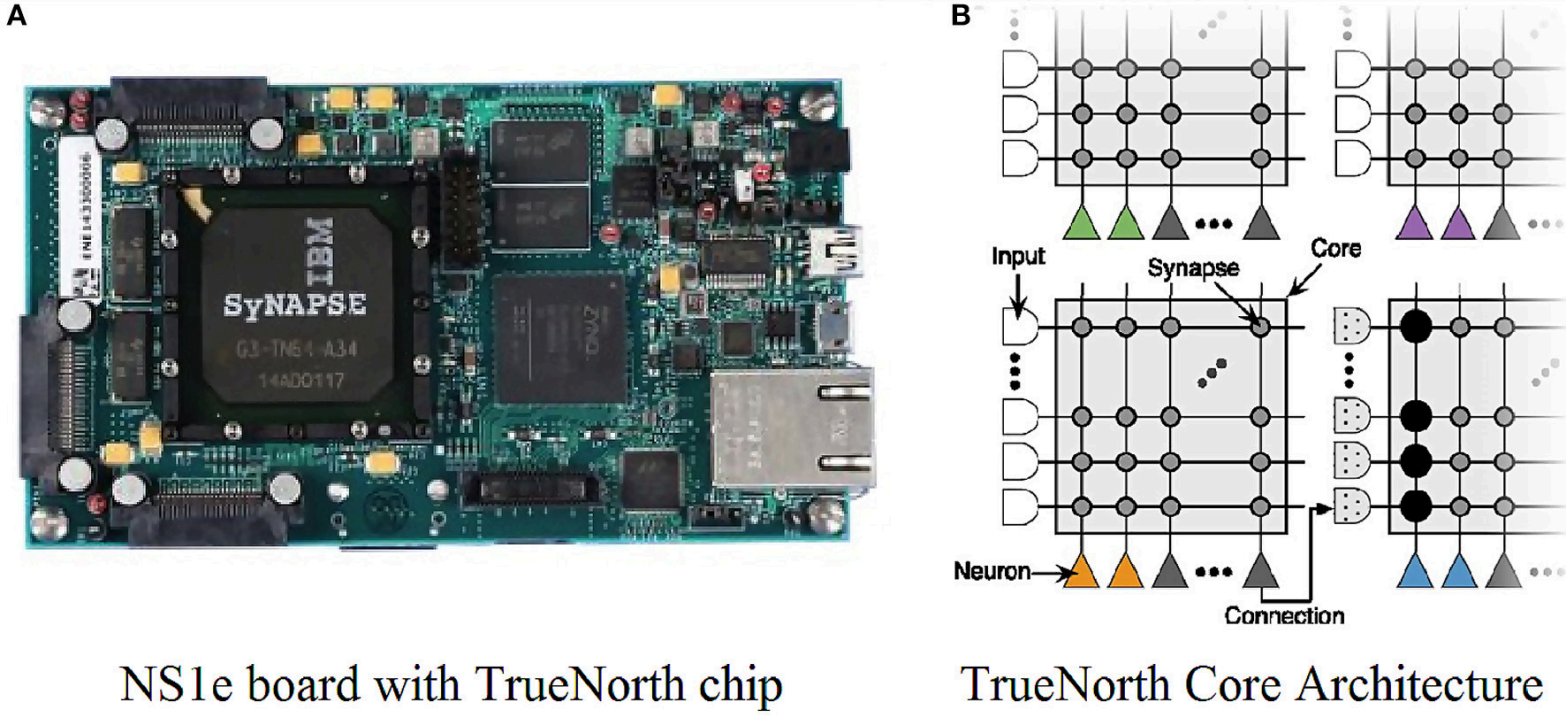
**(A)** IBM TrueNorth chip on NS1e board. **(B)** Each TrueNorth chip has 4,096 cores and each core has 256 input axons, 256 output neurons and 256 × 256 synapses in the connection matrix. Figure adapted from Esser et al. (2016).

The IBM TrueNorth in Figure 2A is a neuromorphic chip with a multicore array of programmable connectivity, synapses and neurons parameters. In our experiment, we used the IBM NS1e board, which contains 4,096 cores, 1 million neurons, and 256 million synapses. TrueNorth has been employed for various convolutional neural network based deep learning applications and serves as ideal hardware for the task of filtering as well.

### 3.1. TrueNorth architecture

TrueNorth chip architecture is organized into programmable neurosynaptic cores and each core consists of 256 incoming input lines mapped to 256 output neurons using a synaptic crossbar as shown in Figure 2B. The crossbar matrix has 256 × 256 programmable synapses and their strength is determined by the corresponding neurons to which they are connected. There are four different axon types that can be assigned to each core's axons, where each core neuron must have identical synaptic weights for synapses connected to axons of the same type. Each synaptic weight is a signed 8-bit value. Each output neuron can connect to the other neurons by projecting its axon (output) to a single input line at the same core or at a different core. Data flow is mediated through spikes either from axons to all the neurons on the same core through binary gate synapses in the crossbar or the neuron itself connects to an axon on the same or a different core. This architecture supports a maximum of 256 incoming axonal inputs for each output neuron and also each input axon can send that input to 256 output neurons on the same core. IBM provides a Corelet Programming Environment (CPE) (Amir et al., 2013) that enables the user to build algorithms that can later be implemented on TrueNorth hardware very effectively. To test various parameters of the axons, neurons and synaptic crossbar connections between axons and neurons, we need to construct a corelet that encapsulates these synaptic cores and their inter-core connectivity. Details of the corelet abstraction and programming are given in Amir et al. (2013)

### 3.2. TrueNorth neuron model

The TrueNorth neuron model is a variation of the “integrate and fire” neuron model with 23 parameters to produce rich neuronal dynamics. The salient features of the membrane potential dynamics of the TrueNorth neuron relevant to this study are captured in Equations (7) and (8).

The update of the neuron membrane potential or the state variable *V*_*j*_(*t*+1) of an output neuron *j* in the next time step (*t*+1) is evaluated by summating the product of incoming synaptic input *S*_*i*_(*t*) from each input axon *i* with the corresponding synaptic weights *w*_*i*_ and adding leak parameter λ_*j*_, and updating in the next time step (*t* + 1) according to the Equation (7). After the update, if the membrane potential exceeds the threshold α_*j*_ of the neuron, then the neuron generates a spike output *OutSpike*_*j*_(*t* + 1) (1 for ON and 0 for OFF). After the spike generation, the membrane potential *V*_*j*_(*t* + 1) resets to reset potential *R*_*j*_ as summarized in Equation (8):

(7) $$\begin{array}{r} V_{j}\left(t+1\right)=V_{j}\left(t\right)+\underset{i}{\sum }S_{i}\left(t\right)w_{i}+\lambda_{j} \end{array}$$

(8) $$\begin{array}{r} if\;V_{j}\left(t+1\right)\geq \alpha_{j},{OutSpike}_{j}\left(t+1\right)=1\;\&\;V_{j}\left(t+1\right)\leftarrow R_{j} \end{array}$$

For comprehensive details about the nomenclature and range of the variables, and various reset mechanisms that are available for TrueNorth neuron model, please refer to Cassidy et al. (2013).

## 4. Noise filtering approach in software and on TrueNorth

**Figure 3:**
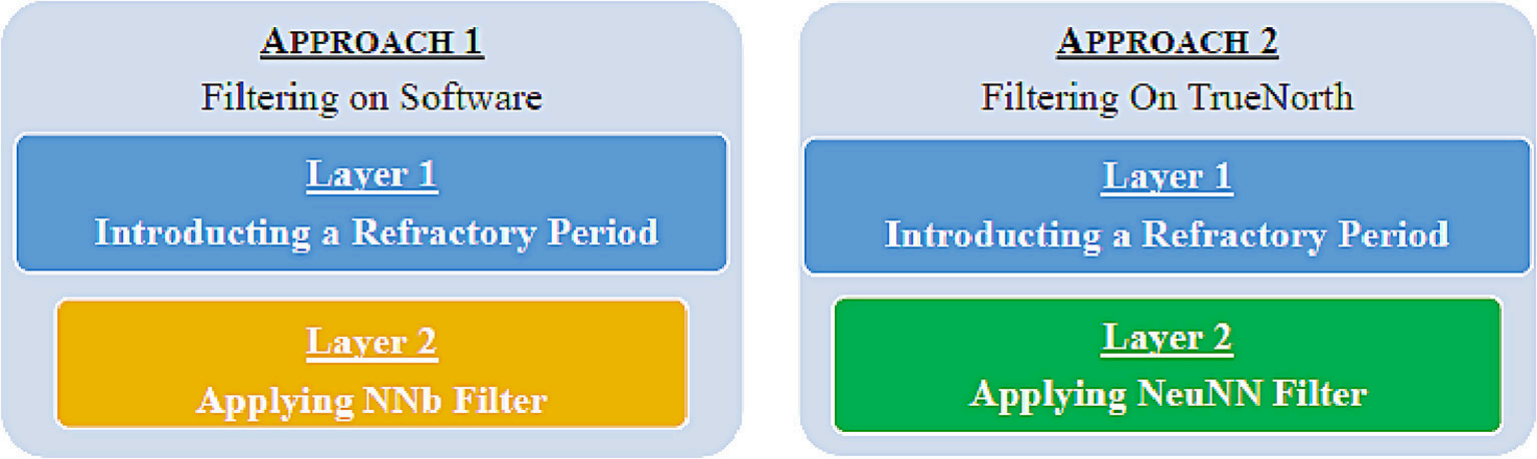
Two layer filtering approaches in software and TrueNorth. The first layer introduces refractoriness and reduces the firing rate of repeatedly firing pixels while the second layer eliminates random noise as they do not have sufficient spatio-temporal support.

Noise in the data from the ATIS recordings has two broad characteristics: (1) high-frequency burst events occur at around 0.7% of the total pixels and (2) low-frequency random events occur throughout the scene. To eliminate both types of noise events, we use a two-layer filtering approach both in software and on TrueNorth as shown in Figure 3. The filtering in software is done to compare the evaluation metrics on the filtered data from software to the filtered data on TrueNorth. This helps us understand the advantage of filtering on TrueNorth both qualitatively and quantitatively. In the first layer filtering on both software and TrueNorth, a refractory period is introduced in the data and the details are discussed in sections 4.1 and 5.1.

There are multiple noise filtering methods for event-based data available in the literature and the Nearest Neighbor (NNb) filter (Dominguez-Morales, 2011; Ieng et al., 2014; Linares-Barranco et al., 2015; Liu et al., 2015) is the most commonly employed filtering method among the other approaches like differing polarity, refractory period and inter-spike interval (Czech and Orchard, 2016). Because the focus of our task is to extract the moving objects and remove the background activity, we look at the NNb filter, which captures the activity of the moving object more aptly. For the second layer filtering on software, the NNb filter is implemented in MATLAB and the description of the algorithm is presented in section 4.2.

For the second layer filtering on TrueNorth, our proposed Neural Network-Based Nearest Neighbor (NeuNN) filtering algorithm, detailed in section 4.3, is used after applying the particular refractory period as the first layer filtering. The NeuNN filtering algorithm is inspired by the Nearest Neighbor filter in Czech and Orchard (2016) and combined with image smoothing operations in conventional image processing (Gonzalez and Woods, 2008). It has been adapted for the TrueNorth hardware to filter the noise from event-based data in real time and the implementation details are discussed in section 5.2.

### 4.1. Layer 1: refractory period filter algorithm

The concept of refractory period draws inspiration from neuronal physiology where a neuron or a cell does not exhibit an immediate response to the stimulus after an action potential and takes a particular duration of time called the “refractory period,” to recover its resources to the normal resting state after an excitation. The refractory period limits the highest frequency of the neuronal spiking and thereby the maximum firing rate.

In the data under study, there are multiple pixels with abnormal firing rates due to the sensor leakage currents at some of the pixels, thus creating high-frequency burst events at these pixels. By applying the refractory period, we introduce a minimum time difference between two consecutive events at a particular pixel and this helps remove the high-frequency sensor noise events more effectively in the next layer on both the NNb filter in software and the NeuNN filter in TrueNorth. The algorithm for applying the refractory period *T*_*ref*_ on the event-based AER data from the ATIS is as follows.

Let the current event received from the image sensor be denoted by *e* = (*x, y, t, p*) and let $T_{\left(x,y\right)}^{last}$ be the time stamp of the last input event at location (*x, y*), independent of polarity that is passed by this filter to the output. Let Δ*T*_(*x,y*)_ denote the time since the last valid event at the location (*x, y*) as given by the following equation:

(9) $$\begin{array}{r} \Delta T_{\left(x,y\right)}=t-T_{\left(x,y\right)}^{last} \end{array}$$

Then, the current event is passed by the filter to the output if and only if the following condition is met:

(10) $$\begin{array}{r} \Delta T_{\left(x,y\right)}\;>\;T_{ref} \end{array}$$

Otherwise, this event is rejected and does not pass through the filter output.

### 4.2. Layer 2 on software: nearest neighbor (NNb) filter

As the name suggests, whenever the *m*^*th*^ event ${e_{\left(x,y\right)}}^{m}$ occurs at any particular location (*x, y*), with the time stamp $T_{\left(x,y\right)}^{m}$, the Nearest Neighbor (NNb) filter considers the time stamp $T_{NNb\left(x,y\right)}^{m}$, of most recent neighborhood event $e_{NNb\left(x,y\right)}^{m}$, excluding the current pixel at (*x, y*) and within a particular distance *D*_(*x,y*)_ surrounding this pixel (*x, y*) in all directions (horizontal, vertical and diagonal). The event is passed through the NNb filter if the time difference between $T_{\left(x,y\right)}^{m}$ and $T_{NNb\left(x,y\right)}^{m}$ is less than the programmable threshold *T*_*NNb*_, i.e.:

(11) $$\begin{array}{r} \text{if}T_{\left(x,y\right)}^{m}-T^{m}<T_{NNb},\text{then event}{e_{\left(x,y\right)}}^{m}\text{at}\left(x,y\right)\text{will be passed} \end{array}$$

The filtered event signifies that the event is associated with neighborhood activity and it is likely related to the moving object. If the time difference is greater than the threshold, then it is more likely that this is a noise event and eliminated through this filter. We will strictly consider the above described NNb filter with distance $D_{\left(x,y\right)}\;\leq \;\sqrt{2}$ (i.e., surrounding eight neighborhood pixels around (*x, y*).

### 4.3. Layer 2 on TrueNorth: neural network based nearest neighbor (NeuNN) filter

As the name suggests, the filter considers the activity or events occurring at that particular pixel location (*x, y*) including the activity at the neighborhood pixels surrounding this pixel (*x, y*). This activity is summed on to a neuron and the output of the activity is determined based on the configuration of the neuronal parameters. The summation of the synaptic activity is combined with the leak and the membrane voltage of the neuron in the previous time step using the neuronal dynamics Equations (7) and (8) given in section 3.2. This summation is compared with the threshold and if it is greater than or equal to the threshold, then the neuron fires a spike. The output of this neuron corresponds to the filtered output of this pixel.

## 5. Implementation of noise filter on TrueNorth hardware

### 5.1. Layer 1: introducing refractory period using the neurons on TrueNorth cores

**Figure 4:**
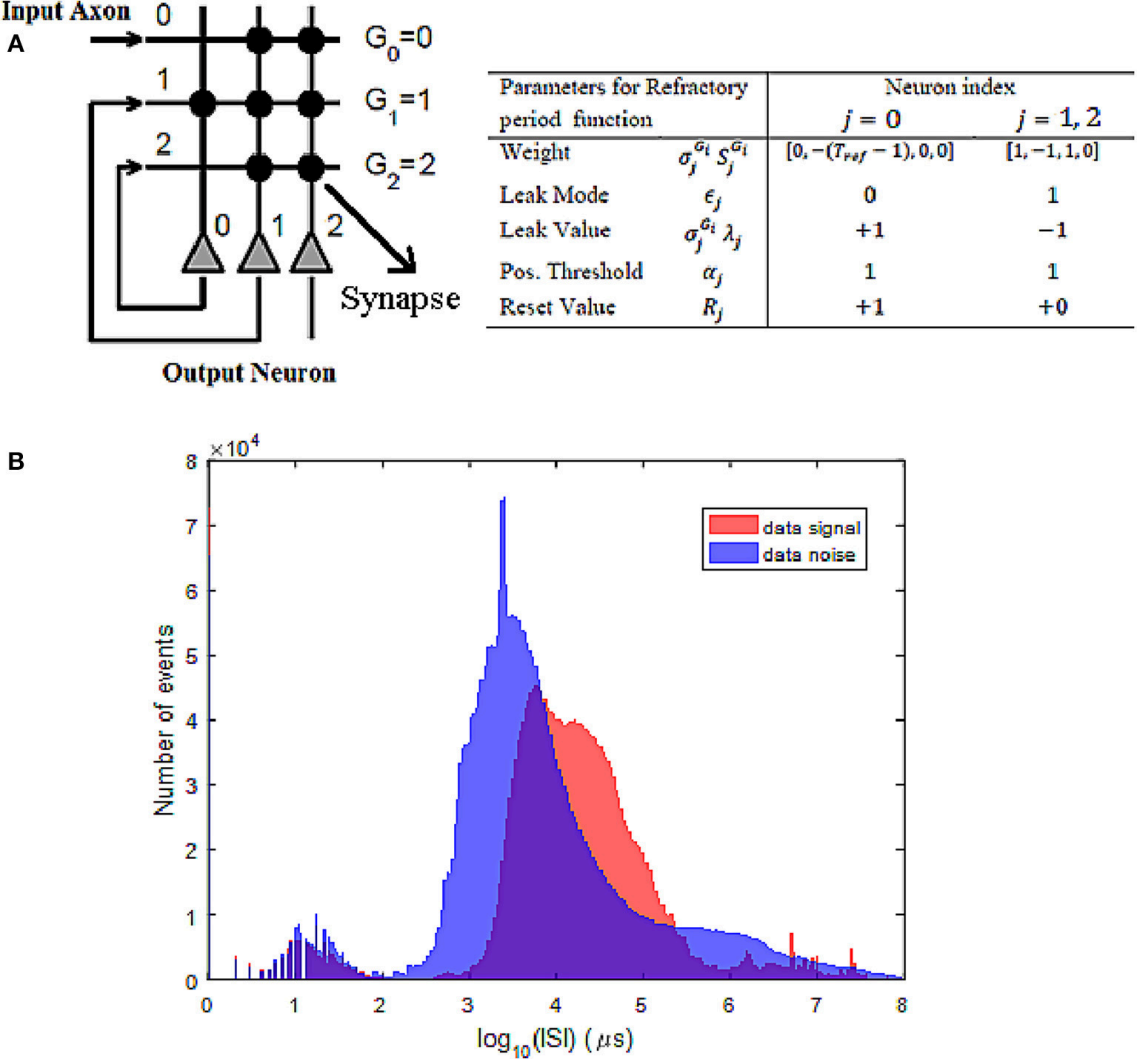
**(A)** Connectivity mapping for refractory neuron function along with the neuronal parameters. **(B)** Statistics of the *ISI* for both signal and noise events show many noise events at *ISI* between 1 and 10 ms, whereas signal events mostly have *ISI* higher than 10 ms. This is used to choose a refractory period value in the range of 3–5 ms.

As described in section 4.1, the refractory period layer incorporates a minimum time difference between the successive events at any pixel location before passing through the NeuNN filter. Each pixel needs three input axons and three output neurons available on the core of the TrueNorth to stream events for the refractory period function. The connectivity mapping and neuronal parameters specified in Figure 4A are used to create the refractory period operation on each input pixel. The output of the neuron labeled 2 produces the spike output with the desired refractory period by configuring the weight of neuron 0. The parameters for neurons 1 and 2 are identical thereby generating multiple copies of the neuronal output. The operation of this circuit may be described as follows: at the start when there is a spike input coming from axon 0, neurons 1 and 2 produce spikes, thereby allowing the first input spike to appear at the output. When there is no spike input, neuron 0 emits a spike every millisecond due to the leak value (+1), threshold value (1) and reset value (+1). However, when there is feedback input coming from the neuron 1 output and it has a negative weight of −(*T*_*ref*_ − 1), it brings down the membrane potential and inhibits the spike, thus providing parameter control to set the desired refractory period. Because there are 256 input axon lines and 256 output neurons in each core, up to 85 pixel inputs can be mapped to each core taking a block of three axons and three neurons for each pixel. To map all the data coming from all pixels of ATIS at least a total of 859 (304 × 240/85) cores are required.

To decide the appropriate setting for *T*_*ref*_, we have plotted the inter-spike interval (*ISI*) histogram (Figure 4B) for both signal and noise events. To do this, signal events were extracted based on annotated tracks of valid objects in the scene while noise events correspond to those occurring outside these marked bounding boxes. It can be seen from Figure 4B that there is as many noise events with *ISI* < 10 ms, whereas most signal events are at *ISI* > 10 ms. To suppress these frequently occurring noise events, a refractory period between 3 and 5 ms seems an appropriate choice.

Based on the above analysis, the refractory period *T*_*ref*_ of 5 ms is used at the first layer of processing the data both on software and TrueNorth, before applying either of the second layer filters such as NNb and NeuNN on software and TrueNorth respectively. There are a total of 96,232,414 events in all the recordings combined and after passing the entire data set through the refractory layer, there is a loss of around 86.6 % of the original events and a total of 12,909,503 events (around 13.4 % of the original events) were retained for the second layer of filtering. The effect of the refractory period on the hardware adaptation of the refractory period layer on TrueNorth is identical to the software implementation with respect to the filtered events and discussed in detail in section 6.

### 5.2. Layer 2: applying NeuNN filter using the neurons on the TrueNorth cores

To map data coming from the ATIS in a regular fashion to each TrueNorth core, we divide the entire frame (304 × 240) into patches of size 12 × 12. Each patch from this is mapped to each core in TrueNorth and this mapping requires a total of 520 cores for mapping all pixels of the ATIS. In this example, we use a 3 × 3 filter size, i.e., for the output neuron at location (*i, j*), we connect it with input pixel (*i, j*) and its eight neighboring pixels. However, the method we describe has also been used to implement 5 × 5 or 7 × 7 filters. All the neurons in this filter use the same parameters of positive and negative reset = 0 and leak mode = 0. For design space exploration, the weight *w* is fixed during the simulation while the other two neural parameters of threshold, α and leak, λ are varied to find optimal parameter sets. This is done because only relative values of (α/*w*) and (λ/*w*) determine the neuronal dynamics and, hence, filtering performance. The details of ATIS input pixels mapping to TrueNorth core are provided in Appendix in Supplementary Material.

## 6. Results and discussion

### 6.1. Layer 2 filter parameter optimization

#### 6.1.1. Software filter (NNb)

For the NNb filter, we swept the value of *T*_*NNb*_ in the range of 0.5–5 ms to observe the effect it has on the three metrics *PSR*^*f*^, *PNR*^*f*^ and *SNR*^*f*^ (in dB). The result of this exploration (on recording at 6:30 p.m.) is shown in Table 4. From Table 4, we can see that *SNR*^*f*^ (in dB) is highest for the smallest value of *T*_*NNb*_ due to the very small amount of remaining noise. However, at this setting, the amount of signal remaining *PNR*^*f*^ is also very low, and in practice, we need a balance between *SNR*^*f*^ (in dB) and *PSR*^*f*^. Hence, in practice, it is better to use values of *T*_*NNb*_ in the range of 1–3 ms. In the rest of the simulations in this paper, we use *T*_*NNb*_ = 1 ms.

**Table 4:** Effect of varying parameter *T*_*NNb*_ in the NNb filter on Filtering Metrics.

***T*_*NNb*_ (ms)**	***PSR*^*f*^**	***PNR*^*f*^**	***SNR*^*f*^**
0.5	11.97	0.09	20.17
1	21.54	0.18	19.74
2	35.36	0.35	19.02
3	44.38	0.51	18.32
4	50.42	0.68	17.69
5	54.49	0.83	17.12

#### 6.1.2. TrueNorth filter (NeuNN)

**Figure 5:**
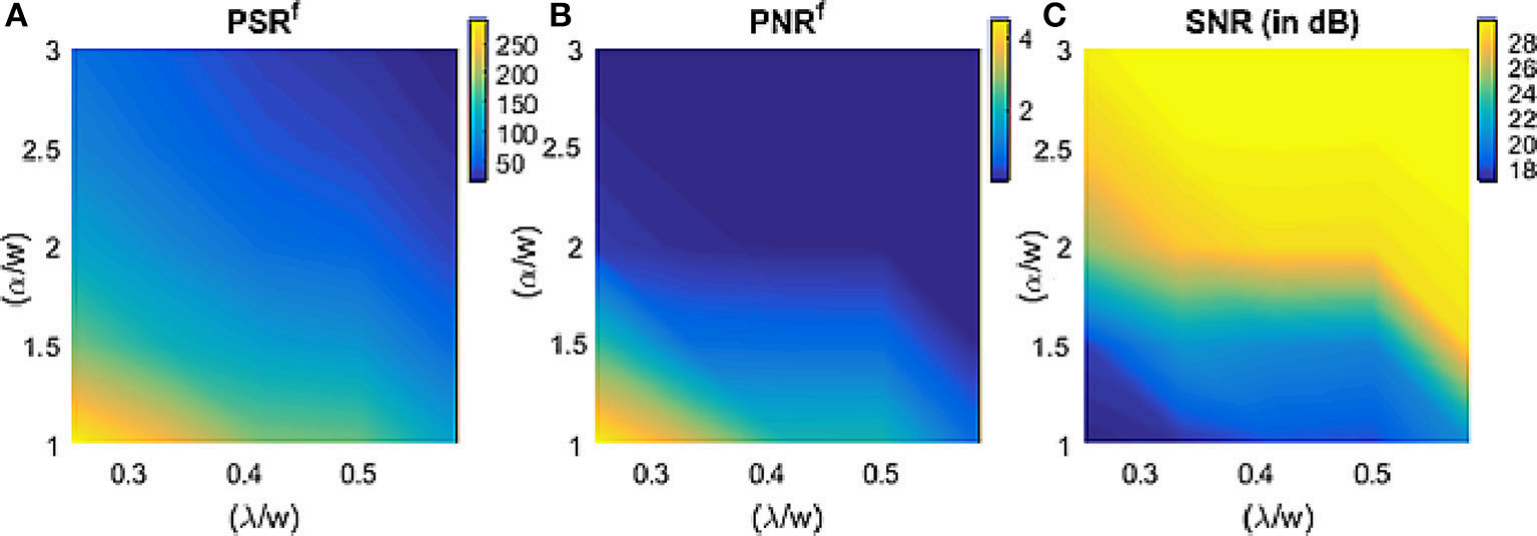
Effect of TrueNorth neuron parameters on the metrics from section 2.4 (the above plot used 6:30 p.m. data).

The NeuNN network is configured with a single type of synapse with synaptic weight *w* for all the synapses and one neuron type with threshold α and leak λ for all the neurons. The design space exploration is carried out with ratio of threshold/weight, (α/*w*) and leak/weight, (λ/*w*) ranging from [1–3] and [0.150–0.7], respectively with a fixed synaptic weight. The filtering metrics presented in section 2.4 are used to study the effect of TrueNorth neuron parameters and the results are plotted in Figure 5.

From Figure 5A, the signal remaining after filtering increases with the decrease of both (α/*w*) and (λ/*w*) and it can be higher than 100%. The reason for this is that the output neurons are receiving inputs from all the neighboring pixels within a particular distance and when (α/*w*) and (λ/*w*) is low, the filter spikes very often, resulting in more output events than input events for the signal. Therefore, instead of any signal loss that typically happens after using traditional filtering methods, we can observe a tremendous amount of signal gain for certain parameter ranges of the NeuNN filter. This behavior can be explained by thinking of NeuNN in this parameter range as a spatiotemporal version of the dilation operator used in traditional image processing (Gonzalez and Woods, 2008). From Figure 5B, we can see that the noise remaining after filtering is also affected similarly as signal remaining with both (α/*w*) and (λ/*w*). Because the objective of this filtering is to keep the noise as low as possible without compromising too much on the signal, we a have a window of parameter space, where (α/*w*) and (λ/*w*) can be configured. It can be seen from Figure 5C that the signal to noise ratio (SNR) increases with both increasing (α/*w*) and (λ/*w*). Choosing the parameters of the filter cannot depend on the high SNR (which is due to the reduction in the noise) alone; the signal remaining and noise remaining both need to be considered in conjunction with the requirements of the following processing steps (Tracking–prefers as low noise as possible and Classification–prefer high amount of signal). We chose two sets of parameters: (1) (α/*w*) = 2 and (λ/*w*) = 0.33, and (2) (α/*w*) = 2.5 and (λ/*w*) = 0.583 where the first set results in higher *PSR*^*f*^ than NNb filter (due to the dilation like behavior described earlier) while the second set results in similar *PSR*^*f*^ as NNb filter. The results in the paper are reported for parameter set 1 by default while we specifically mention when parameter set 2 is used.

### 6.2. Qualitative comparison of both filtering approaches

**Figure 6:**
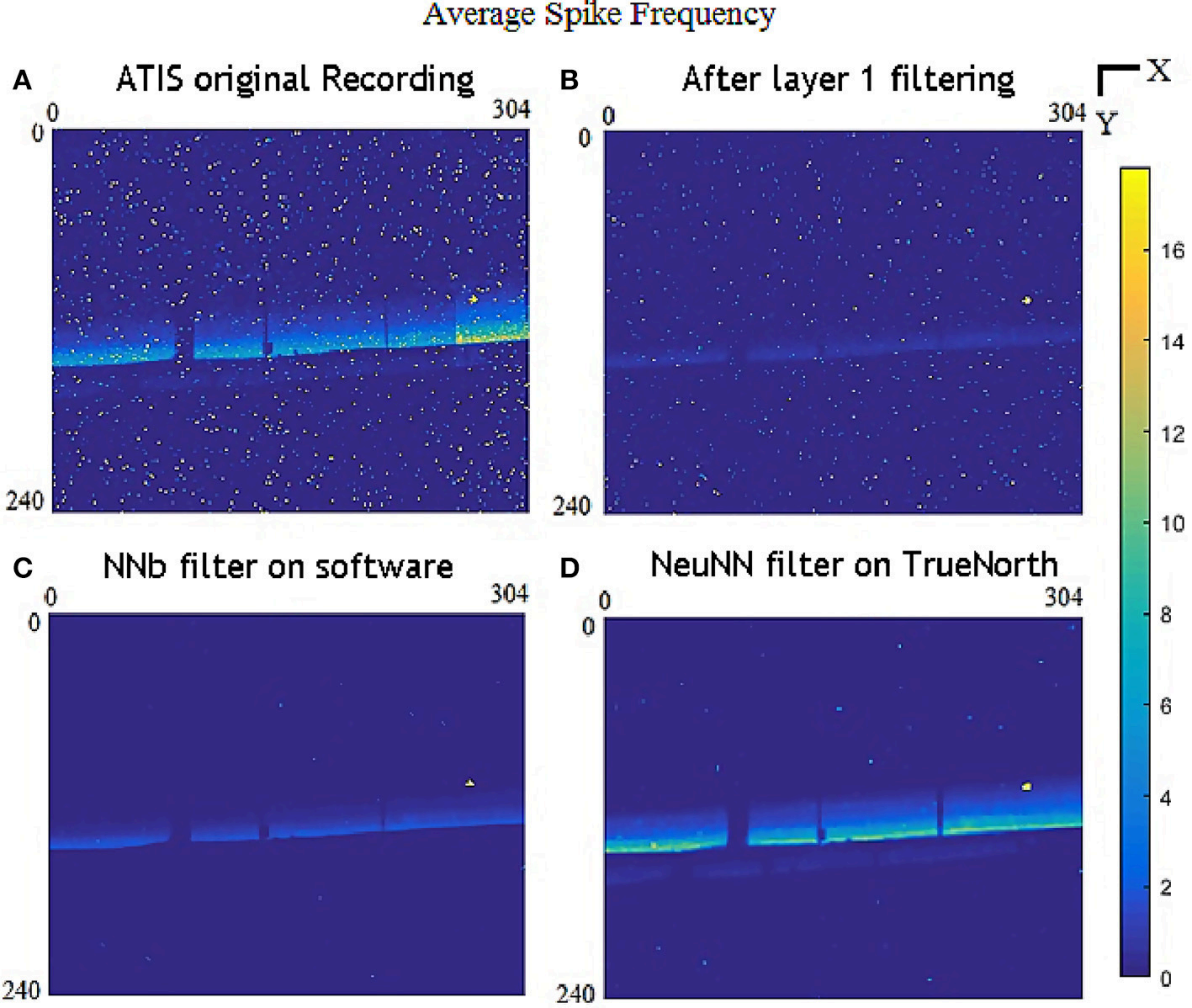
Comparison of color-coded average spike frequency (events/s) at each pixel for all 304 × 240 pixels combining all the sample files for **(A)** original data; **(B)** after layer 1refractory period of 5 ms; **(C)** after layer 1 refractory filter and layer 2 NNb filter on software; and **(D)** after layer 1 refractory filter with layer 2 NeuNN filter on TrueNorth.

From Figure 6, the refractory layer reduces high-frequency temporally localized noise while layer 2 removes spatially spread noise. NeuNN also retains more signal events than the NNb filter. We can notice that the refractory layer significantly reduces the high-frequency noise pixel in yellow.We can also notice the missing activity due to the occlusion of objects and there is a small region of dead pixels (around 8–10 pixels) toward of the right of the frame and the activity is not possible to be removed by both the filters.

**Figure 7:**
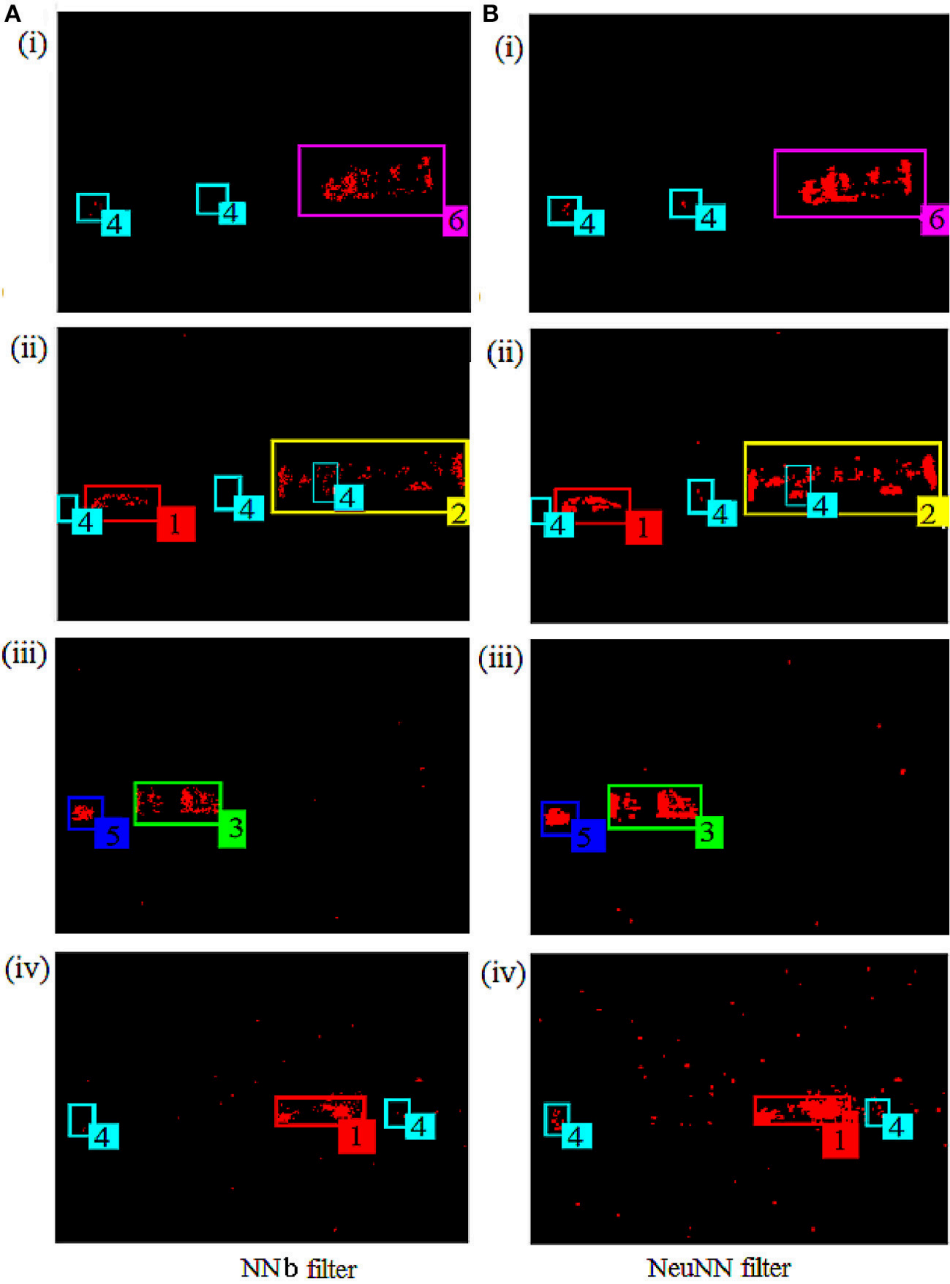
Comparison of NNb filter on software (on the left) with the NeuNN filter on TrueNorth (on the right) using the same time instances and same files mentioned in Figure 1B.

Figure 7 that NeuNN retains many more signal events while keeping slightly more noise. The benefits of NeuNN are most evident for slow-moving objects like pedestrians, where the NNb filter removes almost all signal events. We can visually verify that there are more events in the tracker for all classes of objects in the scene from the NeuNN filter screenshots on the right compared with the NNb filter on the left. Slow-moving pedestrians are the worst performing class in terms of retaining the signal and they are barely visible on NNb filter and this makes them quite challenging to detect and classify. Their activity, however, is visible on the NeuNN filter. There are slightly more noise events present outside the tracker after filtering using the NeuNN filter in comparison with the NNb filter when there is very high back noise activity.

### 6.3. Quantitative comparison of filtering approaches using the metrics

**Figure 8:**
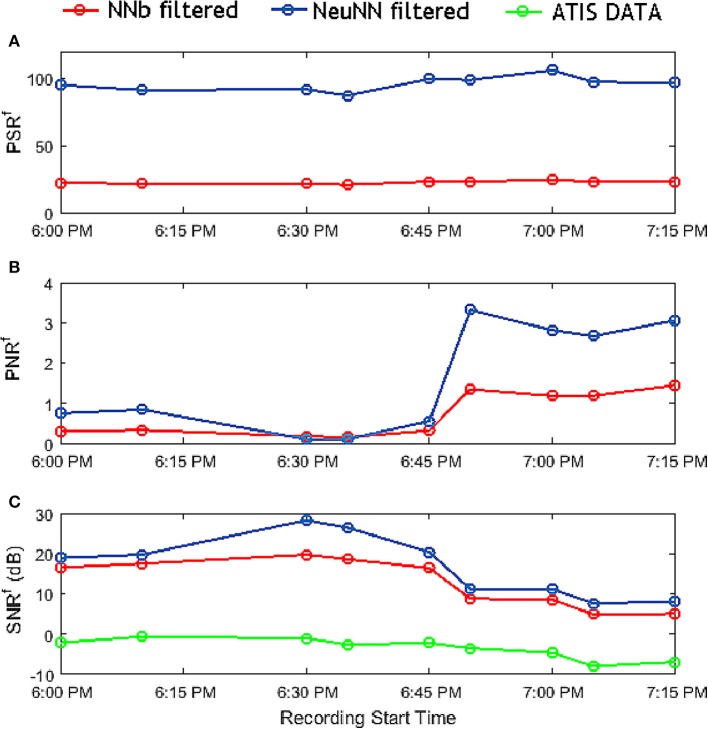
Comparison of all the metrics of NNb filter and NeuNN filter with respect to each sample file at various recording times representing varying background noise characteristics.

Next, we quantitatively evaluate the benefits of the NeuNN filter over the NNb filter using the metrics described earlier. The results are plotted in Figure 8. It can be seen from Figure 8A that the signal remaining after filtering using the NeuNN filter is significantly higher (~3X) compared with the NNb filtering. NeuNN is also capable of producing missing activity in the neighborhoodnone of the traditional filters are capable of doing this. Figure 8B shows that the remaining noise is slightly higher (~2.5X) when the background noise increases significantly at the later part of the night. Finally, Figure 8C shows the SNR before and after filtering. The SNR ratio before and after filtering is compared for the two filtering methods and the SNR (in dB) is significantly higher (by 14.75–29.3 dB) for filtering using NeuNN on TrueNorth due to the retention of the signal after using the neural network-based filter.

**Figure 9:**
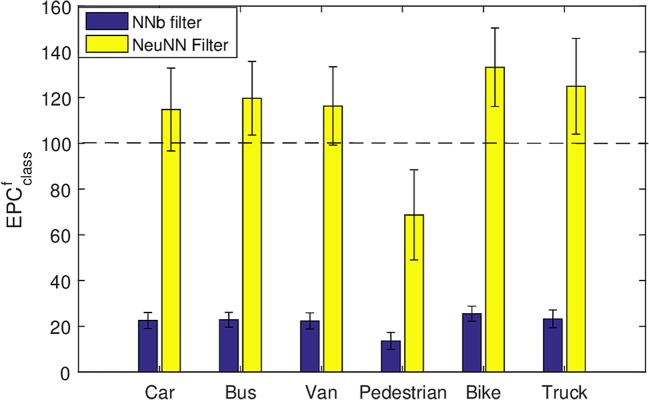
Average percentage of signal events remaining compared with the original data for each class after two-layer filtering using all the samples in Table 1.

A refractory period of 5 ms is used for the first layer filter and the filtering performance for the NNb filter on software and NeuNN filter on TrueNorth are compared. Across all cases, NeuNN generates more than 5X events compared with the NNb filter for all classes of objects moving in the scene. We observe from Figure 9 that the signal loss for the NNb filter is significant for all objects (at around 80%). However, the NeuNN filter generates extra signal events in the case of fast-moving objects (all except pedestrians) compared with the NNb filter. This is a significant advantage of the NeuNN on TrueNorth over the NNb filter. Though slow-moving humans with least spike rate per pixel of 52 spikes/sec (averaged over all videos) are affected the worst in comparison with other objects using both approaches, the signal loss is only around 30% with the NeuNN filter compared with 83% with the NNb filter. This makes it easier to track and classify pedestrians using the NeuNN filter in the later stages of processing. Finally, we observe that the signal remaining is highest for bikes in both approaches. The reason for this is that bikes are compact and fast-moving objects with highest spike rate per pixel of 142 spikes/ sec (averaged across all videos). Other fast-moving objects, like buses, have regions of low contrast (e.g., glass windows) that do not generate events, making their spatial density of events low (approximate spike rate per pixel of 95 spikes/sec averaged across all videos).

### 6.4. Discussion

We have demonstrated a new noise filtering algorithm for event based imagers and shown its implementation on TrueNorth. There are some important points to note about our proposed method. First, it should be noted that since TrueNorth processes data at a millisecond clock tick, we lose the original microsecond resolution of events coming from the sensor. However, for our application of object tracking and classification in traffic monitoring or surveillance, this is acceptable and microsecond temporal resolution is unnecessary.

Second, the response of a rectangular photodiode, such as the ones in ATIS, will be different for horizontally and vertically moving contours (Clady et al., 2017). However, for the traffic monitoring and surveillance applications we are considering, the orientation of the camera will be fixed and we presented results for this orientation. For changes of orientation, our filtering method will still work–however, the exact gains in *SNR*^*f*^ might be different.

Third, we expect the effect of adding more events in regions with signal events (spatio-temporal dilation) will be helpful in tracking under noisy conditions. However, the shape information of the object may be distorted by this filtering. We propose an architecture with two parallel processing paths in that case–the raw signal (or mildly filtered one) goes to a classifier in one path, while in the other path, we have the NeuNN filter followed by the tracker. The tracker informs the classifier about which spatial location to focus on for object classification. In this way, the classifier gets access to raw shape of the object as well as precise location. Also, for applications where the added events are undesirable, we can use other parameters such as parameter set 2 described earlier that has similar *PSR*^*f*^ as NNb filter. Quantitatively, the average values of *PSR*^*f*^, *PNR*^*f*^, and *SNR*^*f*^ for NNb filter are 21.54, 0.18, and ~20dB respectively while the same ones for NeuNN filter with parameter set 2 are 24.04, 0.09, and 29 dB respectively. This shows that even when NeuNN reduces signal similar to levels of NNb filter, it can produce better SNR due to stronger attenuation of noise. For reference, the average values of *PSR*^*f*^, *PNR*^*f*^, and *SNR*^*f*^ for NeuNN with parameter set 1 are 92, 0.11, and 28 dB respectively. Though this setting can address the reduction of dilation effect it cannot ensure complete elimination of this phenomenon and in our envisioned applications it is not a significant issue. However, in applications such as event-based stereovision or event-based optical flow computation (which are principally based on small local spatiotemporal neighborhoods and precise timing) they could have a significant local effect.

## 7. Conclusion and future work

In this work, we presented a novel neural network-based noise filtering (NeuNN) approach and compared it with the typically used nearest neighbor (NNb) noise filter for event-based image sensors. We also described how we can map the NeuNN filter algorithm to TrueNorth architecture efficiently. Though the filter used is available on the TrueNorth platform for implementation, it can be adapted to any other neural network-based neuromorphic hardware for filtering noise from event-based sensors. We showed that the proposed NeuNN filter is capable of generating new events that can be associated with the signal while the output events of traditional filters are strictly a subset of input events. This approach results in much higher signal retention for NeuNN and also results in higher SNR for NeuNN-filtered images.

We used manual annotation information for analysing metrics. In future, we will integrate a tracker and classifier after the filter and evaluate the performance of the filtering algorithm based on tracker and classifier performance. Finally, in the current study, the background noise characteristics change with respect to the time of the recording. When the noise in the environment changes, configuring the neuronal parameters for the task of filtering across varying noise conditions becomes more challenging. To improve the noise filtering performance, a learning mechanism for updating the parameters based on the noise characteristics could be introduced and the possibility of incorporating such a mechanism needs to be studied.

The total number of cores used in this two-layer filtering approach is quite high and increases rapidly with the size of the filter and image. One way to reduce the number of cores is to combine both filtering operations into one layer. In this method, the neurons in the layer 2 filter can be reset to a low value below the threshold and can slowly recover to resting potential through a leak. The time taken by the neuron to recover to its resting potential acts like a refractory period. This method is similar to a relative refractory period (Gerstner and Kistler, 2002) because the neuron can still fire a spike in this period if it receives a large number of input spikes, whereas the method we propose is akin to absolute refractory period. However, this method has the problem that neurons in layer 2 become less excitable in general and do not generate output events even if inputs are coming from surrounding pixels within that refractory period. We found that the percentage of signal that remains after using this approach is less than our proposed solution. Another possibility is to use two layers, but instead of using three neurons per pixel in layer 1 for the refractory period, use one neuron per pixel using the relative refractory method described earlier. However, this still suffers from a non-constant refractory period due to the variable number of spike inputs the neuron receives during the refractory period.

## Author contributions

VP has performed the neural network experiments and simulations on TrueNorth. GO has devised the ATIS setup and collected the data from ATIS. All authors reviewed the manuscript while AB supervised this project.

### Conflict of interest statement

The authors declare that the research was conducted in the absence of any commercial or financial relationships that could be construed as a potential conflict of interest.
