# Supplementary material for: A Noise Filtering Algorithm for Event-Based Asynchronous Change Detection Image Sensors on TrueNorth and Its Implementation on TrueNorth
Source: Front Neurosci. 2018 Mar 5;12:118. doi: 10.3389/fnins.2018.00118 (PMC5844986; doi:10.3389/fnins.2018.00118)
Supplement: Supplementary file 1 [file DataSheet1.pdf]

1 Appendix

1.1 Mapping NeuNN filter to TrueNorth cores

To create a scalable approach to map different filter sizes and image sizes to TrueNorth, we first divide the image into patches of size $M \times M$ for a filter size of $L \times L$ ($L \geq 3$; L is an odd number) such that each patch can be completely processed by one TrueNorth core. In Figure 1, we show this process in detail for $L = 3$; however, in the following text, we will do a general analysis applicable for any filter size. We can divide the patch into an internal smaller patch of size $(M - (L - 1)) \times (M - (L - 1))$ whose pixels do not need to be shared with surrounding patches (shown in yellow in Figure 1B). The remaining boundary pixels are of two types: corner ones, which need to be shared with three surrounding patches (shown in green in Figure 1B), and off-corner ones that need to be shared with one neighboring patch (shown in magenta in Figure 1B).

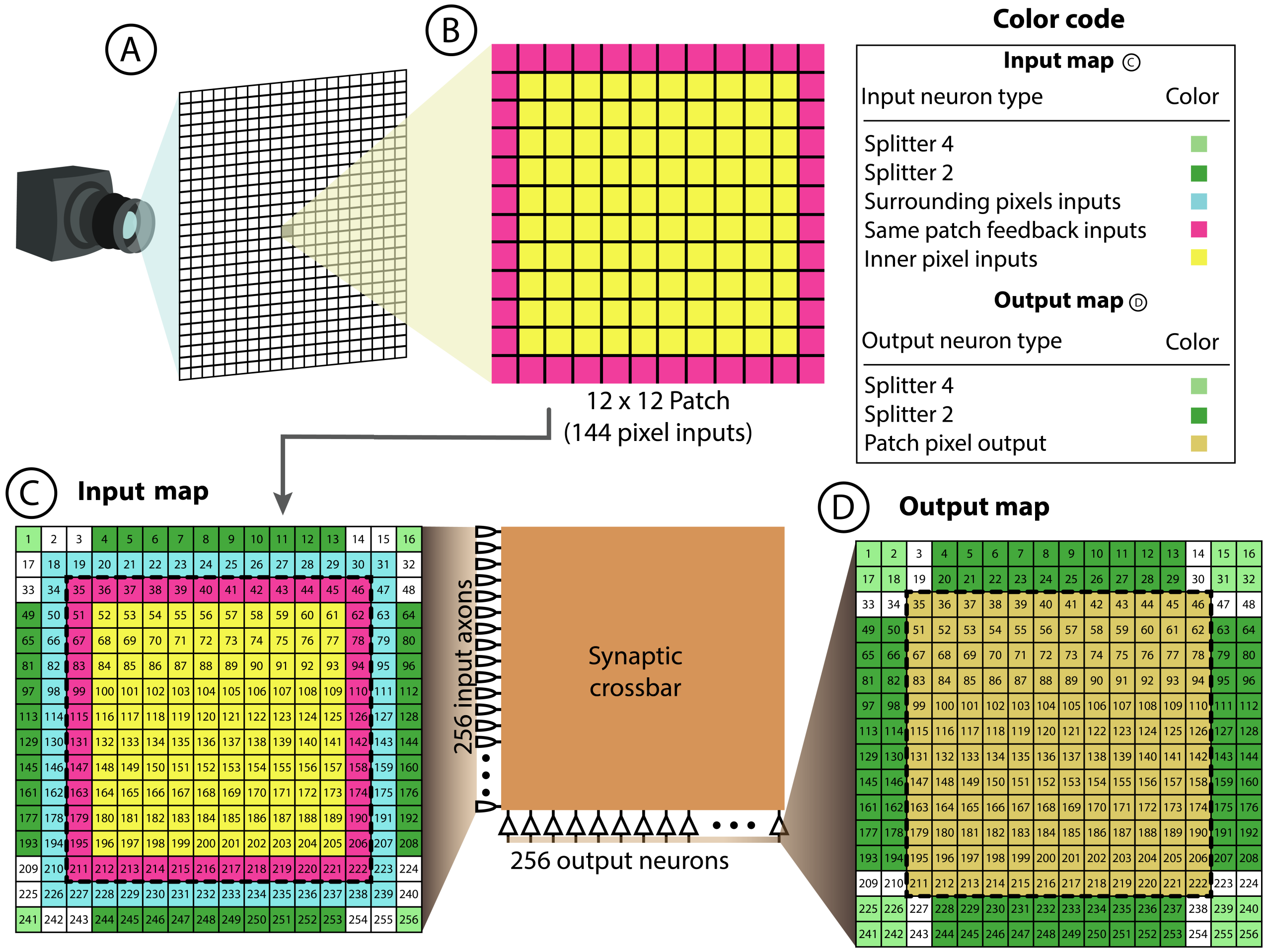

Figure 1: Mapping of ATIS pixels to TN cores for filter size 3×3. (A) The entire pixel plane is divided in to 12×12 patches, which are mapped to a single core on TrueNorth. (B) The boundary pixels of the 12×12 patch need to be connected with neighboring TrueNorth cores as well and, hence, are logically treated separately. (C) The 256 input axons of a core are shown as a 16×16 two-dimensional patch. The yellow axons directly connect with pixels from same patch while pink axons connect to neurons in the same core. These correspond to those boundary pixels of the patch that have to be split into multiple copies by neurons in this core. Blue axons connect to neurons from neighboring cores while green axons connect to input pixels, which need to be split. (D) The 256 output neurons are also shown as a 16×16 patch. The 12×12 biscuit color neurons in the centre produce the 144 filtered outputs of the 12×12 patch mapped to the core while the green neurons at the periphery produce the split outputs to be passed to neighboring cores. The same color coding is used in the picture of input axon mapping to make it clear which output neurons from neighboring cores connect with which input axons.

We refer to the number of neighboring patches connected to this pixel as a share-factor. Hence, internal, off-corner and corner pixels have share-factor equal to 0, 1 and 3, respectively. This also determines the number of neurons needed for each of the pixels as equal to the share-factor + 1 to copy this input as many times as needed. Now, we can quantify the total number of inputs that can be mapped to the 256 available input axons on a TrueNorth core. Denoting the number of axons for internal, off-corner and corner pixels by A_{int}, $A_{off-corner}$ and A_{corner}, respectively, we get:

$$A_{int} = (M - (L - 1))^2 \tag{1}$$

$$A_{off-corner} = 4(L - 1)(M - (L - 1)) \tag{2}$$

$$A_{corner} = 2(L - 1)^2 \tag{3}$$

A_{int} is shown in yellow in Figure 1C. The contribution of $A_{off-corner}$ is split into two colours: the inputs from pixels going to splitter neurons are shown in dark green while the output of the splitter neurons mapped back to this core are shown in magenta. Similarly, for A_{corner}, the inputs from pixels going to splitter neurons is shown in light green while the output of the splitter neurons mapped back to this core are shown in magenta. In addition, just like this core provides inputs to neighbouring cores through splitter neurons, it must also accept inputs from its neighbours. Using A_{neigh} to denote the number of axons connected to splitter neuron outputs from neighbouring cores, we get:

$$A_{neigh} = 2M(L - 1) + (L - 1)^2 \tag{4}$$

A_{neigh} is denoted in blue in Figure 1C. Finally, putting everything together, we get an inequality constraining patch size M as:

$$A_{int} + A_{off-corner} + A_{corner} + A_{neigh} \leq 256 \tag{5}$$

We can write similar equations for the number of output neurons. Using N_{int}, $N_{off-corner}$ and N_{corner} to denote the neurons producing outputs for the patch, splitting off-corner inputs and splitting corner inputs respectively, we get:

$$N_{int} = M^2; N_{off-corner} = 4(L - 1)(M - (L - 1)); N_{corner} = 4(L - 1)^2 \tag{6}$$

These output neurons are shown in Figure 1D (N_{int}, $N_{off-corner}$ and N_{corner} in biscuit color, dark green and light green respectively). Unused neurons and axons are shown in white. Like in the case of axons, we can again have an equation constraining M based on the number of available neurons in a core:

$$N_{int} + N_{off-corner} + N_{corner} \leq 256 \tag{7}$$

Simplifying inequalities (6) and (7), we get the same inequality

$$M^2 + 4M(L - 1) \leq 256; \tag{8}$$

From the above inequality (8), for $L = 3, 5, 7$, maximum M values are $12, 8, 4$ respectively.

1.2 Scaling of Image size and Filter size on True North

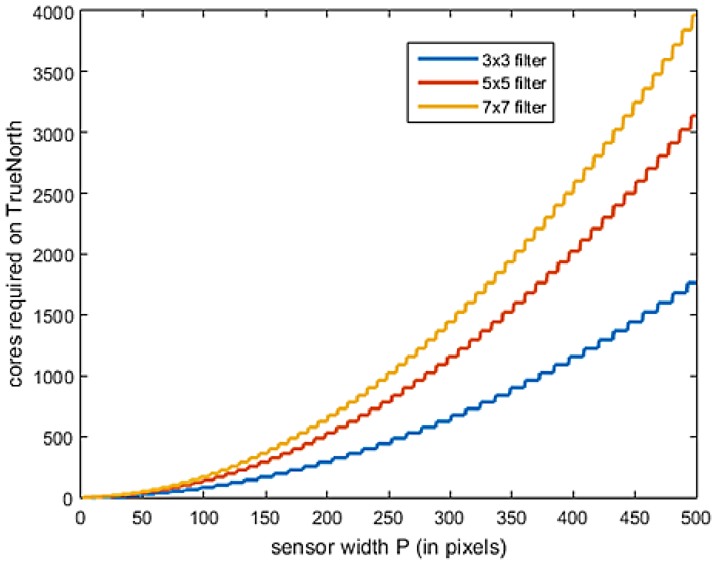

Figure 2: The number of cores required on TrueNorth for an image sensor with resolution $P \times P$ is plotted for different filter sizes. The dependence is quadratic on P and hence proportional to sensor resolution.

For the NeuNN filter, the number of cores required on TrueNorth, N_{Cores}^{NeuNN} for any asynchronous event based sensor of resolution $P \times Q$ where P, $Q \in N$, using filter size of $L \times L$ pixels can be determined by the equation below:

$$N_{Cores}^{NeuNN} = \left\lceil \frac{P}{\lceil \sqrt[2]{4(L-1)^2) + 256} - 2(L-1) \rceil} \right\rceil \times \left\lceil \frac{Q}{\lceil \sqrt[2]{4(L-1)^2) + 256} - 2(L-1) \rceil} \right\rceil \quad (9)$$

where $\lceil x \rceil$ denotes the least integer larger than x and $\lceil \sqrt[2]{4(L-1)^2) + 256} - 2(L-1) \rceil$ is the maximum patch width for a particular filter width L obtained by solving the inequality in (19). The current mapping evaluates $\lceil \sqrt[2]{4(L-1)^2) + 256} - 2(L-1) \rceil \times \lceil \sqrt[2]{4(L-1)^2) + 256} - 2(L-1) \rceil$ neuronal filtering operations per core and for a 3×3 filter, it computes 144 filter operations per core. Figure plots the number of cores for these three filter sizes as a function of image sensor width P, where we assume a square sensor with resolution $P \times P$. As a numeric example, for the 304×240 resolution of the ATIS with a 3×3 filter, 520 cores were utilized, which is approximately one-eighth of the entire TrueNorth chip resources.

1.3 Computational Requirements for Noise Filtering

For the NNb filter, the number of operations involved per event depends on the size of the filter. We need to store an array T_{last} of the same size as the image sensor to keep track of last time an event happened at a particular location. For a $L \times L$ filter, whenever a new event occurs at pixel location (x, y) at time t, $T_{last}(x, y)$ has to be updated to t and this time stamp needs to be compared with the L^2 time stamps from the T_{last} array in its neighbourhood. Hence, the number of operations required to process this event is $L^2 + 1$.

For the NeuNN filter, we consider an optimised FPGA or microprocessor implementation that can be event-driven and not operated on a clock tick like TrueNorth. Here, we need to store two arrays of the size of the image sensor resolution. The first one stores time of last spike T_{last} for the neurons (and not the spike time of input pixel like the earlier NNb filter case) while the second array stores membrane potential V_{mem} of the neuron. For an $L \times L$ filter, whenever a new event occurs at pixel location (x, y) at time t, we need to update the membrane potential due this event at the neighbourhood pixels surrounding this pixel (x, y). We also need to compare each updated V_{mem} with the threshold to determine if a spike is generated. If so, then corresponding

locations of T_{last} have to be updated, leading to the number of operations required per event being between $2L^2$ and $3L^2$. To get an estimate for maximum operating frequency requirement, we have an estimate of maximum average firing rate of 390 keps from our data, where the average is computed over a 1 ms window. Hence, for a 3×3 filter, this would need at most ~10.5 Mops while for a 7×7 filter, the number of operations increases to ~57 Mops.
